# A comparison of traumatic experiences and human rights violations of persons with mental health conditions or psychosocial disabilities and persons with other disabilities

**DOI:** 10.1371/journal.pone.0292750

**Published:** 2023-11-27

**Authors:** Jin Hashimoto, Takashi Izutsu, Shodai Sunagozaka, Satoshi Iiyama, Atsuro Tsutsumi

**Affiliations:** 1 Graduate School of Human and Socio-Environmental Studies, Kanazawa University, Kanazawa, Japan; 2 Graduate School of Agricultural and Life Sciences, The University of Tokyo, Tokyo, Japan; 3 Tokyo Musashino Hospital, Tokyo, Japan; 4 Institute of Transdisciplinary Sciences for Innovation, Kanazawa University, Kanazawa, Japan; 5 University of the Philippines Open University, Los Banos, The Republic of the Philippines; University of Helsinki, FINLAND

## Abstract

This study compares the traumatic and human rights violation experiences of persons with mental health conditions or psychosocial disabilities and those of persons with other disabilities in the Philippines. Additionally, the role of gender in exposure to traumatic experience and human rights protection levels is explored. Of those registered as persons with disabilities in the city of Muntinlupa, 3000 subjects were randomly selected and 1,024 among them (Male = 510, Female = 512, Others = 2) agreed to participate in this study. This comparative study adopts a cross-sectional design. The survey was conducted using a structured questionnaire. The researchers mobilized health workers, officially recruited by the city, to visit the target participants’ houses and to distribute the questionnaires to collect data. The questionnaire comprises items related to the participants’ socio-demographic characteristics, exposure to traumatic experiences, and human rights-based well-being. Regarding the frequency of exposure to traumatic experiences, a little difference was found in physical domestic violence (abuse) between the two groups (Frequency = 20 (9.66), 44 (5.39%), χ2 = 5.154, p < 0.05). Regarding human rights-based well-being, no significant difference was found between persons with mental health conditions or psychosocial disabilities and persons with other disabilities. However, the human rights-based well-being of women with mental health conditions or psychosocial disabilities was significantly worse than that of women with other types of disabilities. Concrete and day-to-day human rights challenges in several areas in addition to inaccessibility to various services may have contributed to the human rights challenges encountered by women with mental health conditions or psychosocial disabilities. De-stigmatization of women with mental health conditions or psychosocial disabilities, the implementation of awareness-raising campaigns on various levels, and developing capacity of women with mental health conditions or psychosocial disabilities on how to protect their own rights may help improve the status quo.

## Introduction

While persons with disabilities constitute a large portion of the world population (15%) [1], they face various forms of human rights violations and continue to be marginalized. Recently, the United Nations (UN) launched the “Disability and Development Report–Realizing the SDGs by, for and with persons with disabilities,” which comprehensively portrays the barriers and human rights violations against persons with disabilities [2]. The efforts of the international community include the adoption of the “Convention on the Rights of Persons with Disabilities” and its Optional Protocol in 2006 [3] and the “Sendai Framework for Disaster Risk Reduction” in 2015, which recognized the importance of inclusive policies for persons with disabilities [4]. The disability perspective was further incorporated into the Sustainable Development Goals (SDGs) in 2015 [5]. Despite this progress, human rights violations against persons with disabilities still persist.

Among persons with disabilities, persons with mental health conditions or psychosocial disabilities (MHPD) are of particular concern as they face multiple forms of human rights violations. For example, 37% of countries restrict the right to marry for persons with MHPD [6], and a vast majority of countries (approximately 90%) even restrict their right to vote [7]. Further, women with MHPD may be at a particular disadvantage as they are disproportionally affected by varied human rights violations including sexual violence [8].

Despite the grim challenges faced by persons with MHPD, there have been few studies that quantitatively describe their human rights situations or compare them with persons with other types of disabilities. Crucially, there have been a handful of studies comparing women with MHPD with women with other disabilities, despite the fact that being a girl or a woman in addition to being a person with MHPD may present unique and intersecting challenges.

To review a limited number of previous studies, Hughes et al. [9] indicated in their systematic review and meta-analysis that persons with MHPD might be at significant risk of being victimized by violence. However, the authors indicate that there is not enough evidence on the prevalence and risk of violence against persons with disabilities and that conducting further research would be helpful. In addition, the authors pointed out that most studies have been conducted in developed countries, leaving developing countries underrepresented.

Khalifeh et al. [10] investigated data gathered from 44,398 adults through the British Crime Survey and made comparisons among persons without disabilities, persons with MHPD, and persons with disabilities other than MHPD. Through their large-scale study, they revealed that persons with MHPD experience the highest risk of violence and that they are three times more likely to be victims of past-year violence than persons without disabilities. However, the study’s primary focus was on violence and did not examine the overall human rights situation of persons with disabilities.

Based on a Danish national survey of over 18,000 citizens, Dammeyer and Chapman [11] revealed that persons with MHPD have a strong tendency to become targets of violence and discrimination compared to persons with physical disabilities. A study that investigated the prevalence of disability-based discrimination and the related health impacts in Australia articulated that disability-based discrimination leads to psychological distress and deterioration of self-rated health and that persons with intellectual disabilities tend to report disability-based discrimination more often [12]. Another Australian quantitative study analyzed discrimination experienced by persons with different types of disabilities and their avoidance behavior due to their disabilities [13]. The study found that psychosocial or physical disabilities are significant factors that increase the possibility of facing discrimination or experiencing avoidance behavior. However, these studies have some limitations. Concerning the Danish study, the question posed to the participants to determine whether they suffered discrimination was simplified. Therefore, the details of the discrimination experienced are unclear. Likewise, the question asked to determine the presence of discrimination in Australian studies was also genericsimple, and the specifics of discrimination were not described sufficiently. It would be difficult to formulate concrete policies to tackle disability-based discrimination without data on the specifics of the experience.

Notably, women with MHPD are more likely to be victimized by sexual violence and harassment in comparison to women without disabilities, which is reported by numerous studies including those by Khalifeh et al. [14] and Kamperman et al. [15]. At the same time, studies describing different human rights-related realities of women with MHPD, and women with other disabilities are not much prevalent.

To the best of our knowledge, the scarcity of large-scale studies quantitatively investigating human rights situations can be attributed to the fact that sophisticated tools to quantify human rights conditions were not available. However, to formulate appropriate policies to prevent discrimination and human rights violations, it is vital to accurately grasp the present situation by conducting analyses on detailed data collected with a sophisticated tool. In this regard, the application of the tool to comprehensively quantify human-rights protection levels by examining various day-to-day situations can be considered one of the most significant strengths of the present study.

Further, it is estimated that 80% of the one billion persons with disabilities live in developing countries, and the percentage of people aged 60 and above with disabilities in lower-income countries (43.4%) is much higher than that in higher-income countries (29.5%) [1]. Given the overwhelming number of persons with disabilities and low-resource settings in developing countries, conducting a large-scale study in a developing country and analyzing realities faced by both men and women with MHPD is crucial.

Therefore, this study examines the human rights situations of, and traumatic experienced by persons with MHPD and persons with other disabilities in the Philippines and compares the two with a gender perspective. The present study further investigates whether gender plays a role in the difference in exposure to traumatic experience and in human rights protection levels. In other words, the present study aims to (1) quantify human rights situations and exposure to traumatic events and (2) determine whether there are any differences in terms of human rights situations as well as exposure to traumatic events between persons with MHPD and persons with other disabilities in the Philippines.

## Methods

### Participants

This study was conducted between January 2019 and January 2020 in the city of Muntinlupa, which is located in the southernmost part of Metro Manila, the capital city area of the Philippines. According to the Persons with Disability Affairs Office, Social Affairs Bureau, the Government of the City of Muntinlupa, 11,586 individuals were registered as persons with disabilities in the city as of May 2021. A total of 1,024 persons with disabilities participated in our study. In terms of the selection procedure, we stratified sample size based on the types of disabilities (persons with MHPD and persons with disabilities other than MHPD) and used a power of 90%, a 1:4 group ratio, and a significance level of 0.05. We estimated the mean score of the Human Rights-based Well-being Checklist (HRWC) of persons with MHPD at 93.6 (60% of the perfect score) with a Standard Deviation (SD) of 30 and the mean score of HRWC of persons with disabilities other than MHPD at 109.2 (70% of the perfect score) with SD of 20. In the first group, we estimated a sample size of 45, and in the second group, we estimated a sample size of 180. However, given the scarcity of studies quantitatively analyzing the levels of human rights protection, we hypothesized that it would be necessary to at least have a fourfold sample size (1,000) to maintain good precision and adequate power. Therefore, we randomly approached 3,000 individuals out of the individuals registered as persons with disabilities, assuming that two-thirds of them would not be able or willing to participate. Health workers officially employed by the city of Muntinlupa with clinical expertise and sensitivity to issues concerning disabilities visited the participants’ households to collect data. Excluding participants for reasons, such as refusal to participate in the study, being away from home at the time of our visit, and inability to answer our questions owing to the nature of the disabilities, we obtained a total of 1,024 responses. As a result, the actual power reached a sufficient 86.7%. All the participants voluntarily participated in the study without compensation.

In the city of Muntinlupa, types of disabilities are officially categorized into six categories: (1) mental and psychosocial disabilities, (2) communication disabilities, (3) learning disabilities, (4) intellectual disabilities, (5) orthopedic disabilities, and (6) visual disabilities. According to the categorization system of the city of Muntinlupa, if an individual has more than one type of disability, then that individual is categorized as a person with the leading type of disability, and therefore, no one is registered as a person with multiple disabilities. The registration of persons with disabilities is conducted based on the clinical judgment of clinicians, such as medical doctors.

### Instruments

This comparative study adopts a cross-sectional design. To assess the exposure to human rights violation and traumatic experiences among persons with MHPD as well as persons with disabilities other than MHPD, a structured questionnaire was employed in each interview. The questionnaire included questions concerning socio-demographic characteristics, exposure to traumatic experiences, and human rights-based well-being.

In terms of socio-demographic characteristics, participants data on age, gender, religion, job status, and family income were collected. Regarding exposure to traumatic experiences, participants were asked to indicate whether they had suffered (1) physical domestic violence (abuse), (2) verbal domestic violence (abuse), (3) sexual violence (abuse), or (4) witnessed something, and we compared the frequency of exposure to different traumatic experiences (i.e. the number of persons with MHPD as well as the number of persons with other disabilities exposed to different traumatic experiences). To assess and quantify human rights-based well-being, the HRWC was administered. The HRWC, which was developed based on the Convention on the Rights of Persons with Disabilities (CRPD) in collaboration with the United Nations, disability experts, and organizations of persons with disabilities, among others, is a tool to “quantify and compare states of protection of rights among individuals and specific groups” [16]. The HRWC contains 39 questions that assess the degree to which people have experienced certain human rights violations in the last one year in various day-to-day situations. The validity and reliability were confirmed (α = 0.90). Participants answer each question on a scale of 0 to 4, and some questions need to be scored in reverse, necessitating score adjustment. The total score is calculated between 0 and 156, and the higher the score, the higher the human rights-based well-being. HRWC has been developed in such a way that it can be used in developing countries including the Philippines [16].

### Data analysis

The socio-demographic characteristics (age, gender, religious affiliation, employment status, and family income) of the participants were analyzed using mean, frequency, and median. The chi-square test was used to compare the categorical variables, that is gender, religious affiliation, and employment status of the two groups; t-test was used to analyze the age differences between the two groups, while and Mann-Whitney U-test was used to compare the family income differences between the two groups. The chi-square test was conducted with information on the frequency of exposure to different traumatic experiences to make a comparison between persons with MHPD and persons with disabilities other than MHPD in terms of traumatic exposure, and a t-test was performed to compare the two groups’ HRWC scores. IBM SPSS Statistics (Version 27) was used for the analyses, and the statistical significance was set at 0.05.

### Ethical considerations

Substantial precautions were taken owing to the sensitive subject matter. All potential participants received written information in simple language about the purpose of the study, methods, voluntariness of participation, and the right to withdraw at any time. With assistance from local health workers who had received two days of extensive training on research ethics, informed consent forms were read aloud to ensure that potential participants could make a voluntary choice regardless of their literacy level. All participants except for illiterate individuals provided written informed consent. Illiterate individuals provided verbal informed consent. Ethical approval was obtained from the Ethical Committee of Kanazawa University (approval number: KINDAIKOKUKI-001GO). The Muntinlupa City Health Office approved the study protocol in advance.

## Results

### Socio-demographic characteristics

Of the 1,024 persons with disabilities who participated in our study, 207 were persons with MHPD (108 males, 98 females, 1 other). The mean age of persons with MHPD was 34.87 years (SD = 12.78). The number of persons with disabilities other than MHPD was 817 (102 persons with communication disabilities, 35 persons with learning disabilities, 30 persons with intellectual disabilities, 568 persons with orthopedic disabilities, and 82 persons with visual disabilities); among them, 402 are males, 414 are females, and 1 other. The mean age of persons with disabilities other than MHPD was 40.92 (SD = 13.99).

As shown in Table 1, significant differences were observed in terms of age between persons with MHPD and persons with disabilities other than MHPD (the former were younger) (p < 0.01). No significant differences were observed in the gender composition between the two groups.

**Table 1:** Socio-demographic characteristics of participants (Persons with MHPD and persons with disabilities other than MHPD).

	Persons with MHPD Total (n = 207)		Persons with Disabilities other than MHPD Total (n = 817)		t^a^/χ^2b^/U^c^
	Mean^a^/ Frequency^b^/ Median^c^	SD^a^/%^b^/ 1st-3rd quartile^c^	Mean^a^/ Frequency^b^/ Median^c^	SD^a^/%^b^/ 1st-3rd quartile^c^	
Age^a^	34.87	(12.78)	40.92	(13.99)	-5.658**
Gender^b^					1.759
Male	108	(52.17)	402	(49.20)	
Female	98	(47.34)	414	(50.67)	
Other	1	(0.48)	1	(0.12)	
Religion^b^					16.877**
Christianity	153	(73.91)	687	(84.09)	
Islam	9	(4.35)	9	(1.10)	
Others	42	(20.29)	113	(13.83)	
No Belief in Any	3	(1.45)	8	(0.98)	
Job Status^b^					0.204
Employed	58	(28.02)	242	(29.62)	
Not Employed	149	(71.98)	575	(70.38)	
Monthly Family Income^c^ (PHP)	10000	(6000–15000)	10000	(6000–15810)	83.589
Types of Disabilities Registered^b^					
Mental and Psychosocial	207	(100.00)	0	(0.00)	
Communication	0	(0.00)	102	(12.48)	
Learning	0	(0.00)	35	(4.28)	
Intellectual	0	(0.00)	30	(3.67)	
Orthopedic	0	(0.00)	568	(69.52)	
Visual	0	(0.00)	82	(10.04)	

^a^ t-test

^b^ χ^2^ analysis

^c^ Mann-Whitney U-test

* p < 0.05

** p < 0.01

Note: If a person has more than one type of disability, then that person is categorized as a person with the leading type of disability.

In terms of religious beliefs, out of 207 persons with MHPD, 153 believed in Christianity (73.91%), 9 believed in Islam, 42 had other religious beliefs, and 3 did not have any religious beliefs. Of the 817 persons with disabilities other than MHPD, 687 believed in Christianity (84.09%), 9 believed in Islam, 113 had other religious beliefs, and 8 did not have any religious beliefs. As a result, significant statistical differences were observed between the two groups (p < 0.01).

Regarding employment status, 58 persons with MHPD were employed at the time of the survey (28.02%) and 149 were unemployed (71.98%). Likewise, 242 persons with disabilities other than MHPD were employed at the time of the survey (29.62%) and 575 were unemployed (70.38%). No significant differences were observed between the two groups in terms of employment.

In terms of the annual family income of the household to which the selected participants belonged, the median income was PHP 120,000 (PHP 10,000 × 12 months) for both groups. Therefore, no significant differences were observed between the two groups in terms of annual family income.

### Traumatic exposure

Table 2 shows the frequency of exposure to different traumatic experiences (i.e. the number of persons with MHPD as well as the number of persons with other disabilities exposed to different traumatic experiences). In terms of exposure to “physical domestic violence (abuse),” there was significant differences between persons with MHPS and persons with other disabilities. On the other hand, there were no significant differences between persons with MHPD and persons with other disabilities in terms of exposure to the other traumatic experiences (i.e. verbal domestic violence (abuse), sexual violence (abuse), witnessed something and any trauma).

**Table 2:** Frequency of exposure to different traumatic experiences.

	Persons with MHPD Total (n = 207)		Persons with Disabilities other than MHPD Total (n = 817)		χ^2^
	Frequency	%	Frequency	%	
Physical domestic violence (abuse)	20	(9.66)	20	(5.39)	5.154*
Verbal domestic violence (abuse)	28	(13.53)	95	(11.63)	0.563
Sexual violence (abuse)	11	(5.31)	31	(3.79)	0.97
Witnessed something	7	(3.38)	37	(4.53)	0.528
Any Trauma	53	(25.60)	169	(20.69)	2.353

Chi-square test * p<0.05

** p<0.01

Note: If a person has more than one type of disability, then that person is categorized as a person with the leading type of disability.

Table 3 shows the frequency of exposure to different traumatic experiences (i.e. the number of persons with MHPD as well as the number of persons with other disabilities exposed to different traumatic experiences) disaggregated by gender. In terms of exposure to “physical domestic violence (abuse),” there were no significant differences between the men and women in the two groups, respectively although persons with MHPD as a whole (men and women combined) were more exposed to physical domestic violence (abuse) than persons with disabilities other than MHPD as a whole (p < 0.05) as is indicated above.

**Table 3:** Frequency of exposure to different traumatic experience disaggregated by gender.

	Male					Female				
	Persons with MHPD (n = 108)		Persons with Disabilities other than MHPD (n = 402)		χ2	Persons with MHPD (n = 98)		Persons with Disabilities other than MHPD (n = 414)		χ2
	Frequency	%	Frequency	%		Frequency	%	Frequency	%	
Physical domestic violence (abuse)	10.00	4.83	24.00	2.94	1.48	9.00	4.35	20.00	2.45	2.81
Verbal domestic violence (abuse)	17.00	8.21	48.00	5.86	1.11	11.00	5.31	46.00	5.63	0.00
Sexual violence (abuse)	3.00	1.45	15.00	1.84	0.23	7.00	3.38	16.00	1.96	1.99
Witnessed something	3.00	1.45	16.00	1.96	0.34	4.00	1.93	21.00	2.57	0.17
Any Trauma	28.00	13.53	83.00	10.16	1.39	24.00	11.59	85.00	10.40	0.74

Chi-square test * p<0.05, ** p<0.01

Note: If a person has more than one type of disability, then that person is categorized as a person with the leading type of disability.

In terms of exposure to “verbal domestic violence (abuse),” “sexual violence (abuse),” and “witnessed something,” there were no significant differences between men or women in the two groups or between persons with MHPD as a whole and persons with disabilities other than MHPD as a whole.

Finally, to examine exposure to any traumatic experience (i.e., the percentage of persons who experienced at least one form of traumatic event out of the four types of traumatic events described above), there were no significant differences between the men and women in the two groups, respectively. Likewise, there were no significant differences between persons with MHPD as a whole (men and women combined) and persons with disabilities other than MHPD as a whole.

In short, the exposure to physical domestic violence (abuse) by persons with MHPD as a whole and by persons with disabilities other than MHPD as a whole was the only element where significant differences were observed.

### Human rights-based well-being

Table 4 shows the perceived status of protection of rights, or human rights-based well-being, calculated by the HRWC among persons with MHPD and persons with disabilities other than MHPD. We found that there were significant differences in the total HRWC scores (p < 0.01) between the two groups. In terms of each item in the HRWC, significant differences were observed between the two groups in questions 1, 2, 4, 9, 23, 24, 25, 28, 34, 36 and 39, and for all the items, the scores of persons with MHPD were lower than their counterparts. In order to further analyze the data with an attention to gender, we prepared the Table 5 which displays 39 questions and the total score of the HRWC disaggregated by gender.

**Table 4:** Comparison between persons with MHPD and persons with disabilities other than MHPD for Human Rights-based Well-being Checklist (HRWC).

		Persons with MHPD (n = 207)		Persons with Disabilities other than MHPD (n = 817)		*t* ^*a*^	
		Mean	SD	Mean	SD		
1	I am suffering from inequality & discrimination due to my sex/gender.	3.18	1.23	3.40	1.05	2.32	*
2	I am suffering from inequality & discrimination due to my age.	3.07	1.34	3.38	1.11	3.02	**
3	I am suffering from inequality & discrimination due to my disability.	2.95	1.36	3.13	1.18	1.83	
4	I am suffering from inequality & discrimination due to my race or ethnicity.	3.24	1.35	3.51	1.05	2.68	**
5	I am respected as a capable person.	2.28	1.43	2.42	1.43	1.22	
6	I can move around in this city without much barriers.	2.07	1.42	2.12	1.45	0.44	
7	I can use necessary transportation to participate in social life.	1.95	1.34	2.07	1.44	1.11	
8	I can access necessary information to participate in social life.	2.22	1.31	2.35	1.34	1.32	
9	I can access internet and other new technologies.	2.06	1.45	2.38	1.41	0.86	**
10	I can ask for support from others when needed.	2.48	1.23	2.44	1.23	-3.93	
11	I can participate in decision-making related to my community and nation if I want.	1.87	1.38	2.03	1.38	1.48	
12	I feel threats to my life in my daily life.	2.53	1.31	2.51	1.33	-0.15	
13	I think I can protect my minimum safety when disasters happen in this city.	1.90	1.36	2.08	1.36	1.70	
14	My legal decision-making will be obstructed by others.	2.62	1.41	2.61	1.35	-0.05	
15	I can protect my money and assets without interference from others.	2.06	1.48	2.28	1.43	1.94	
16	I can access legal services such as the court when needed.	2.08	1.44	1.96	1.45	-1.08	
17	I can be detained unlawfully.	3.00	1.40	3.16	1.29	1.48	
18	I can be subject to physical or sexual violence.	3.09	1.32	3.21	1.25	1.25	
19	I can make decisions on my body and mind without interference from others.	2.17	1.48	2.46	1.40	2.60	
20	I can decide where to live.	2.30	1.50	2.60	1.44	2.65	
21	My place of living is OK.	2.78	1.25	2.96	1.23	1.90	
22	I can make my opinion heard when needed.	2.31	1.37	2.60	1.31	2.81	
23	I can make my opinion heard when needed.	2.37	1.48	2.59	1.39	2.98	*
24	I can participate in fair election.	2.32	1.53	2.61	1.53	2.45	*
25	I can marry and have a child without much interference from others, if I want.	2.29	1.55	2.69	1.46	3.44	**
26	I can communicate with my family when I want to.	3.00	1.23	3.12	1.14	1.35	
27	I can communicate with my friends when I want to.	2.57	1.34	2.75	1.20	1.74	
28	I can have/had quality education.	2.23	1.46	2.45	1.36	2.07	*
29	I can receive necessary health services when I would have a physical health condition.	2.63	1.23	2.66	1.20	0.27	
30	I can receive necessary health services when I would have a mental health condition.	2.57	1.22	2.60	1.20	0.34	
31	I think I can receive necessary social services to live in community when needed.	2.54	1.19	2.54	1.21	0.03	
32	I can choose my work if I want to.	2.08	1.41	2.21	1.44	1.19	
33	My work environment is/would be OK (If I’d work).	2.22	1.49	2.41	1.48	1.67	
34	My standard of living (food, clothing and housing) is adequate.	2.66	1.27	2.87	1.20	2.28	*
35	I have access to clean water and toilet.	2.93	1.20	3.08	1.13	1.74	
36	I can access/enjoy culture and the arts (including music, films, theatres, museums, libraries, etc.)	2.36	1.42	2.59	1.31	2.11	*
37	I can access/enjoy sports.	2.18	1.39	2.23	1.41	0.46	
38	I can enjoy tourism and leisure when I want to.	2.16	1.30	2.36	1.33	1.92	
39	I have freedom to keep my culture and religion.	2.58	1.33	2.95	1.23	3.54	**
	Total Score	95.89	27.07	102.37	26.83	3.10	**

^a^ t-test

**Table 5:** Comparison between persons with MHPD and persons with disabilities other than MHPD for Human Rights-based Well-being Checklist (HRWC) disaggregated by gender.

		Men						Women					
		Persons with MHPD (n = 108)		Persons with Disabilities other than MHPD (n = 402)		*t* ^*a*^		Persons with MHPD (n = 98)		Persons with Disabilities other than MHPD (n = 414)		*t* ^*a*^	
		Mean	SD	Mean	SD			Mean	SD	Mean	SD		
1	I am suffering from inequality & discrimination due to my sex/gender.	3.05	1.34	3.37	1.11	2.34	*	3.35	1.10	3.43	0.99	0.75	
2	I am suffering from inequality & discrimination due to my age.	2.95	1.48	3.34	1.14	2.54	*	3.21	1.17	3.42	1.07	1.64	
3	I am suffering from inequality & discrimination due to my disability.	2.85	1.47	3.04	1.24	1.25		3.07	1.20	3.22	1.12	1.18	
4	I am suffering from inequality & discrimination due to my race or ethnicity.	3.21	1.43	3.51	1.05	2.00	*	3.27	1.27	3.52	1.04	1.85	
5	I am respected as a capable person.	2.33	1.39	2.34	1.43	0.07		2.23	1.48	2.48	1.43	1.53	
6	I can move around in this city without much barriers.	2.20	1.41	2.00	1.40	-1.32		1.94	1.43	2.23	1.50	1.73	
7	I can use necessary transportation to participate in social life.	2.06	1.29	1.95	1.43	-0.76		1.85	1.37	2.19	1.45	2.12	*
8	I can access necessary information to participate in social life.	2.24	1.27	2.27	1.35	0.21		2.20	1.36	2.43	1.33	1.53	
9	I can access internet and other new technologies.	2.11	1.39	2.29	1.38	1.17		2.01	1.52	2.46	1.43	2.78	**
10	I can ask for support from others when needed.	2.55	1.18	2.39	1.24	-1.18		2.42	1.28	2.49	1.23	0.50	
11	I can participate in decision-making related to my community and nation if I want.	1.93	1.39	1.88	1.38	-0.29		1.81	1.38	2.17	1.36	2.37	*
12	I feel threats to my life in my daily life.	2.40	1.37	2.52	1.26	0.90		2.69	1.22	2.50	1.39	-1.34	
13	I think I can protect my minimum safety when disasters happen in this city.	1.94	1.34	1.97	1.36	0.18		1.85	1.40	2.19	1.35	2.24	*
14	My legal decision-making will be obstructed by others.	2.56	1.43	2.58	1.34	0.10		2.68	1.39	2.65	1.36	-0.22	
15	I can protect my money and assets without interference from others.	2.10	1.47	2.14	1.38	0.26		2.01	1.51	2.41	1.47	2.38	*
16	I can access legal services such as the court when needed.	2.11	1.48	1.83	1.41	-1.83		2.06	1.41	2.09	1.49	0.17	
17	I can be detained unlawfully.	3.08	1.35	3.19	1.24	0.77		2.93	1.45	3.13	1.34	1.32	
18	I can be subject to physical or sexual violence.	3.11	1.34	3.23	1.22	0.89		3.07	1.32	3.19	1.27	0.85	
19	I can make decisions on my body and mind without interference from others.	2.14	1.48	2.30	1.37	1.07		2.19	1.50	2.60	1.41	2.56	*
20	I can decide where to live.	2.33	1.49	2.45	1.48	0.73		2.26	1.54	2.74	1.38	2.89	**
21	My place of living is OK.	2.95	1.13	2.85	1.28	-0.86		2.61	1.34	3.08	1.17	3.19	**
22	I can make my opinion heard when needed.	2.36	1.30	2.43	1.32	0.49		2.27	1.45	2.76	1.28	3.09	**
23	I can make my opinion heard when needed.	2.36	1.30	2.43	1.32	0.45		2.38	1.43	2.74	1.39	2.28	
24	I can participate in fair election.	2.44	1.47	2.49	1.54	0.35		2.20	1.59	2.72	1.51	0.12	**
25	I can marry and have a child without much interference from others, if I want.	2.20	1.53	2.53	1.49	2.04	*	2.40	1.56	2.85	1.42	2.60	*
26	I can communicate with my family when I want to.	3.09	1.11	3.11	1.14	0.12		2.92	1.35	3.13	1.14	1.46	
27	I can communicate with my friends when I want to.	2.69	1.25	2.75	1.18	0.49		2.47	1.43	2.76	1.22	1.85	
28	I can have/had quality education.	2.25	1.47	2.39	1.35	0.92		2.20	1.46	2.51	1.37	1.96	*
29	I can receive necessary health services when I would have a physical health condition.	2.78	1.10	2.55	1.21	-1.77		2.47	1.35	2.76	1.18	1.99	*
30	I can receive necessary health services when I would have a mental health condition.	2.69	1.13	2.49	1.19	-1.53		2.43	1.30	2.70	1.19	1.98	*
31	I think I can receive necessary social services to live in community when needed.	2.64	1.09	2.47	1.23	-1.32		2.42	1.29	2.61	1.20	1.38	
32	I can choose my work if I want to.	2.13	1.42	2.01	1.44	-0.76		2.01	1.40	2.40	1.42	2.47	*
33	My work environment is/would be OK (If I’d work).	2.40	1.49	2.27	1.50	-0.77		2.02	1.49	2.54	1.45	3.18	**
34	My standard of living (food, clothing and housing) is adequate.	2.71	1.22	2.81	1.22	0.76		2.61	1.33	2.93	1.18	2.16	*
35	I have access to clean water and toilet.	3.00	1.10	3.01	1.16	0.10		2.85	1.31	3.15	1.09	2.14	*
36	I can access/enjoy culture and the arts (including music, films, theatres, museums, libraries, etc.)	2.37	1.41	2.55	1.33	1.21		2.37	1.44	2.63	1.30	1.78	
37	I can access/enjoy sports.	2.39	1.37	2.18	1.43	-1.38		1.97	1.37	2.29	1.39	2.03	*
38	I can enjoy tourism and leisure when I want to.	2.31	1.30	2.29	1.34	-0.10		2.01	1.30	2.42	1.33	2.77	**
39	I have freedom to keep my culture and religion.	2.76	1.25	2.90	1.26	1.02		2.40	1.41	2.99	1.21	3.85	**
	Total Score	99.79	24.42	98.12	25.95	0.48		94.10	29.71	105.52	27.36	3.65	**

^a^ t-test

* p<0.05

** p<0.01

Note: If a person has more than one type of disability, then that person is categorized as a person with the leading type of disability.

Although there were no significant differences between the total HRWC scores of the men in the two groups, there were significant differences in the total HRWC scores (p < 0.01) between the women in the two groups. The significant differences in the female population led to significant differences between persons with MHPD as a whole (men and women combined) and persons with disabilities other than MHPD as a whole (p < 0.01).

On examining each question in the HRWC, there were four questions out of the 39 questions that yielded significant differences between males with MHPD and males with disabilities other than MHPD, and for all four questions, the score was lower for males with MHPD. There were 23 questions that yielded significant differences between women with MHPD and women with disabilities other than MHPD, and for all the 23 questions, the score was lower for women with MHPD. The number of questions where significant differences were observed among female participants was almost six times larger than the number of those where significant differences were observed among male participants.

## Discussion

In terms of demographic characteristics, significant differences were not observed between the two groups in terms of gender, employment status, and annual family income. Although significant differences were observed between the two groups in terms of age and religion, the present study showed that the economic conditions of the two groups were relatively at the same level. According to the Philippine Statistical Authority, the median annual family income in the Philippines is PHP 203,000 [17] (Philippine Statistical Authority, 2018). Since the median annual family income of both households of persons with MHPD and households of persons with disabilities other than MHPD is PHP 120,000 (PHP 10,000 × 12 months), the economic conditions of persons with disabilities, regardless of whether they have MHPD or other disabilities, are highly challenging (less than 60% of the median annual family income of the entire population).

In terms of exposure to traumatic events, exposure to physical domestic violence (abuse) by both groups as a whole (men and women combined) was the only area where significant differences were observed. This means that the two groups were similarly exposed to other forms of traumatic events and that the percentage of persons who experienced at least one form of traumatic event did not differ statistically between the two groups.

The reason for similar levels of traumatic exposure between the two groups may be explained by several factors. First, owing to the invisibility of MHPD, persons with MHPD may not necessarily be a greater target of violence than persons with disabilities other than MHPD. This invisibility might have served as a factor that did not increase the odds of traumatic exposure of persons with MHPD. Second, violence against persons with disabilities as a whole may be pervasive, regardless of the type of disability. It may be the case that stigma and discrimination targeting persons with disabilities as a whole are overwhelming. Third, the number of traumatic events, whether physical domestic violence (abuse), verbal domestic violence (abuse), sexual violence (abuse), or witnessing something, may increase after major disasters because “disasters and extreme weather events directly and indirectly affect the enjoyment of a range of human rights, including the right to life, water and sanitation, food, health, housing, self-determination and culture, as well as the right to development” [18]. The Philippines was ranked third in the world for highest disaster risks, and the risk is “largely due to the location and geographical context as the risk involving coastal hazards such as typhoons, storm surges, and rising sea levels is high” [19]. Therefore, anyone can be victimized regardless of the types of disabilities that they possess, and vulnerability to natural disasters and hazards may be a factor that homogeneously affects all persons with disabilities and make them equally prone to traumatic events.

At the same time, traumatic exposure alone does not determine the level of overall well-being and human rights situation, and in terms of human rights-based well-being, the scores of the HRWC among women with MHPD were significantly lower than those of women with disabilities other than MHPD (no statistically significant differences were observed between the total scores of male persons in the two groups).

The reasons behind the lower HRWC scores among women with MHPD can also be explained from several perspectives. First, women with MHPD face a number of challenges in terms of their human rights in everyday situations. This is reflected by the fact that 23 out of 39 questions in the HRWC yielded significantly lower scores for women with MHPD, demonstrating pervasive human rights violations against them. Second, women with MHPD may experience more concrete challenges than their male counterparts. Examining the four questions that yielded significantly lower scores of male persons with MHPD, it seems that these questions contain rather abstract contents such as “I am suffering from inequality & discrimination due to my age” and “I am suffering from inequality & discrimination due to my race or ethnicity.” In contrast, human rights violations experienced by women with MHPD include items such as “I can use necessary transportation to participate in social life” and “I have access to clean water and toilet,” demonstrating that they face various basic human rights violations to fulfill their basic needs. This discussion is certainly not to underestimate the seriousness of human rights violations experienced by male persons with MHPD, but to help understand the nature of human rights violations experienced by persons of both genders. Third, women with MHPD may have limited access to health services compared to their male counterparts (see No.29/30 of HRWC as examples) because they may be even more stigmatized than their male counterparts. The stigma placed upon them may discourage women with MHPD from seeking support and health services out of fear that they may be discriminated against and treated unfairly by service providers.

These are the possible reasons among others for the significantly lower scores of women with MHPD. Their significantly lower scores led to significantly lower scores for persons with MHPD as a whole (men and women combined) than persons with disabilities other than MHPD as a whole.

The present study had some limitations. First, this study was conducted in only one city in the Philippines, located in the southernmost part of Metro Manila, and rural populations did not participate in the study. Therefore, while the present study is valuable in that it thoroughly analyzed human rights situations surrounding persons with disabilities in urban settings, conducting further research in rural areas would be beneficial.

Second, because of the categorization system of the city of Muntinlupa, no person was identified as having multiple types of disabilities. Therefore, the present study was not able to depict the realities of persons with multiple disabilities concerning human rights situations. There is a possibility that the findings of the present study underestimated the severity of human rights violations against persons with multiple disabilities, as it may be more prevalent and serious. Further, in the categorization system of the city of Muntinlupa, persons with disabilities other than MHPD, include persons with communication disabilities, learning disabilities, and intellectual disabilities. However, communication disabilities, learning disabilities, and intellectual disabilities are closely associated with or some may even think of them as sub-categories of MHPD. This categorization system might have affected the result.

Third, disclosure bias could have affected the results of traumatic exposure. Although the study by Goodman et al. [20] showed that information on trauma history and PTSD obtained from persons with serious MHPD is largely reliable, Khalifeh et al. [10] suggested that persons with disabilities may underreport cases of violence because of potential negative consequences of disclosure or their reliance on perpetrators. In addition, some may refrain from reporting traumatic experiences out of fear that reporting those experiences could trigger negative emotions. Therefore, traumatic cases may have been underreported.

Further, there is a possibility that cognitive impairments related to MHPD might affect the outcomes.

## Conclusion

This is the first study to quantify human rights situations using the scale and examine exposure to traumatic experiences in low-and middle-income countries. The present study revealed that the human rights situations of women with MHPD are considerably worse than those of women with disabilities other than MHPD. Concrete, intersecting and day-to-day human rights challenges on several occasions, coupled with inaccessibility to various services, may have contributed to the results. Being a person with MHPD, who has historically been neglected and marginalized, in addition to being a woman, means dual marginalization and is often associated with stigma. Further research on the associated factors of severe human rights violations against women with MHPD is warranted using detailed statistical analysis in the future.
